# Daily behavioral profiles and academic underperformance among university students: a cross-sectional study

**DOI:** 10.3389/fpsyg.2026.1856492

**Published:** 2026-09-11

**Authors:** Yue Xu, Siyu Zhao, Yilin Zhu, Wenlong Du, Chuanyuan Wang, Shuhan Man, Shuyi An, Chu Zheng, Ping Zeng

**Affiliations:** 1 School of Life Sciences, Xuzhou Medical University, Xuzhou, Jiangsu, China; 2 Department of Biostatistics, School of Public Health, Xuzhou Medical University, Xuzhou, Jiangsu, China; 3 School of Pharmacy, Xuzhou Medical University, Xuzhou, Jiangsu, China; 4 First School of Clinical Medicine, Xuzhou Medical University, Xuzhou, Jiangsu, China; 5 Jiangsu Key Laboratory of Geriatric Precision Medicine and Aging Intervention, Department of Biostatistics, Jiangsu Engineering Research Center of Biological Data Mining and Healthcare Transformation, Xuzhou Medical University, Xuzhou, Jiangsu, China

**Keywords:** academic performance, daily routines, Firth logistic regression, Gower dissimilarity, partitioning around medoids, university students

## Abstract

**Background:**

Daily routines may be associated with university students’ academic outcomes, but isolated-exposure analyses can obscure how behaviors co-occur. We examined associations between learning and lifestyle behaviors and academic underperformance and explored multivariable behavioral profiles.

**Methods:**

This cross-sectional study included 322 first- to third-year life-sciences undergraduates. The primary outcome was academic underperformance, defined as grade point average (GPA) < 2.00. Exposure-specific and primary multivariable associations were estimated using Firth penalized logistic regression. Breakfast frequency and breakfast timing were modeled separately because of structural overlap. Mixed ordinal and categorical behaviors were clustered using Gower dissimilarity and partitioning around medoids (PAM); *k* = 2–6 were evaluated by silhouette width and 200 repeated 80% subsamples.

**Results:**

Academic underperformance occurred in 97 students (30.12%). In the primary frequency model, compared with eating breakfast every day, eating breakfast sometimes (adjusted odds ratio [aOR] = 7.37, 95% confidence interval [CI]: 1.65–70.32) and never eating breakfast (aOR = 30.89, 95% CI: 6.00–317.35) were associated with higher odds of underperformance. Gaming for 4–6 h/day (aOR = 5.58, 95% CI: 2.36–14.19) or >6 h/day (aOR = 13.70, 95% CI: 3.46–60.29), compared with <2 h/day, showed similar associations. Formal interaction tests by sex and academic year were not significant. PAM identified a Dysregulated Routine profile (*n* = 182) and a Structured Routine profile (*n* = 140); underperformance prevalence was 47.8% and 7.1%, respectively. GPA was excluded from cluster formation.

**Conclusion:**

Lower breakfast frequency and prolonged gaming remained associated with academic underperformance, and the two routine profiles differed markedly in academic outcomes. These exploratory, cross-sectional findings require replication in prospective cohorts before they can be translated into student-support strategies.

## 1 Introduction

Academic performance in higher education reflects cognitive preparation together with behavioral, psychological, and environmental influences (Alshareef et al., 2024; Bardach et al., 2023; Cerni et al., 2021; Lozano-Blasco et al., 2022; Stenson et al., 2021; Wei et al., 2021; Zhang et al., 2025). A large meta-analysis of university students has shown that academic achievement is associated not only with traditional cognitive factors but also with motivation, self-regulation, learning strategies, personality, and psychosocial context (Richardson et al., 2012). For university students, lower GPA may co-occur with disrupted daily rhythms, limited self-regulated study, psychological distress, and uncertainty about professional identity (Ahmady et al., 2021; Beiter et al., 2015; Scherrer and Preckel, 2021; Wei et al., 2021; Zou et al., 2024). These factors are potentially relevant to student support, but observational associations should not be treated as causal effects or as validated individual-level predictions.

Sleep timing, breakfast consumption, smartphone use, gaming, and study routines have each been associated with cognition or academic achievement (Altaf et al., 2022; Bouchefra et al., 2023; Neuman et al., 2024; Ramos-Vallecillo et al., 2024; Reuter et al., 2021; Whatnall et al., 2024). Recent systematic reviews and meta-analyses further support associations of sleep quality, breakfast skipping, and technology-related behaviors with academic performance, although effect sizes and study designs vary considerably across the literature (Kuş, 2025; Seoane et al., 2020; Seura et al., 2025). Most studies, however, evaluate behaviors separately. Because daily behaviors are correlated and often measured as ordered or nominal categories, analyses need both exposure-specific models and methods that can describe co-occurring mixed-data patterns without using the academic outcome to construct those patterns.

Life-sciences undergraduates face combined coursework, laboratory training, and professional-development demands (Lee and Chiu, 2022; Seo et al., 2021). Recent systematic evidence in health-professions education indicates that effective behavioral regulation, time management, sustained effort, and strategic learning are consistently associated with academic success (Campione et al., 2026). A transparent definition of academic underperformance and robust estimation are particularly important when some behavior categories contain few outcome events. Conventional maximum-likelihood logistic regression may produce unstable or infinite estimates under separation, whereas penalized likelihood can provide finite estimates while retaining all prespecified categories.

We therefore examined first- to third-year life-sciences undergraduates with three aims: (1) to estimate associations of learning and lifestyle behaviors with academic underperformance, defined consistently as GPA < 2.00; (2) to evaluate whether the main behavior associations differed by sex or academic year using formal interaction tests; and (3) to identify exploratory routine profiles using a mixed-data clustering method and assess their stability. The primary GPA threshold was distinguished from a secondary empirical bottom-quartile indicator, and the analyses were framed as association and exploratory profiling rather than causal or predictive modeling.

## 2 Materials and methods

### 2.1 Study design and participants

A cross-sectional survey was conducted on 22 May 2025 among first- to third-year undergraduates in the School of Life Sciences at a medical university in China. Participants were recruited using one-stage cluster random sampling, with classes as the sampling units. Eligible first- to third-year classes were randomly selected, and all eligible students in the selected classes were invited to participate. Thirteen classes were selected, including four from the 2022 cohort, four from the 2023 cohort, and five from the 2024 cohort. Among 378 eligible students invited from these classes, 322 completed the questionnaire and were included in the final analysis, yielding a response rate of 85.2%. Electronic questionnaire responses were linked to academic performance using student numbers as described in the informed-consent process. The final analytical sample included 168 female students (52.17%) and 154 male students (47.83%); 95 freshmen (29.50%), 110 sophomores (34.16%), and 117 juniors (36.34%). All 322 records had complete values for the variables used in the analyses.

### 2.2 Questionnaire and variables

The original survey was an investigator-developed, self-administered questionnaire covering demographic characteristics, learning behaviors, psychological and emotional experiences, daily routines, consumption patterns, and career planning. Most items were standalone categorical or ordinal questions assessing specific behaviors or circumstances rather than components of a single psychometric scale; therefore, internal-consistency measures such as Cronbach’s alpha were not considered the primary measurement property for these items.

For the present study, variables relevant to learning behaviors and daily routines were selected according to the prespecified research objectives, including sex, academic year, study-related behaviors, sleep, breakfast habits, mobile-phone use, gaming duration, and selected demographic and professional-attitude variables. Psychological and emotional items were not included in the primary analyses. Original response categories were retained where analytically appropriate, while adjacent categories were combined for selected variables to reduce sparsity and improve interpretability. The analytic categories used in the present study are reported in Table 1 and Supplementary Table 1. A mapping of the original questionnaire response options to the analytic categories is provided in Supplementary Table 6.

**TABLE 1:** Selected participant characteristics by academic-underperformance status (GPA < 2.00).

Characteristic	Category	Total *n*	GPA < 2.00, *n* (%)	*P*
Sex	Female	168	35 (20.8)	<0.001
	Male	154	62 (40.3)	
Academic year	Freshman	95	32 (33.7)	0.007
	Sophomore	110	42 (38.2)	
	Junior	117	23 (19.7)	
Library use (h/week)	>15 h	38	1 (2.6)	<0.001
	7–15 h	80	9 (11.2)	
	<7 h	204	87 (42.6)	
Online study (h/week)	>3 h	62	2 (3.2)	<0.001
	1–3 h	159	45 (28.3)	
	<1 h	101	50 (49.5)	
Timing of first laboratory entry	Freshman	90	9 (10.0)	<0.001
	Sophomore	62	5 (8.1)	
	Junior	12	2 (16.7)	
	Not yet entered	158	81 (51.3)	
Review initiation	Continuous	19	4 (21.1)	<0.001
	1 month	90	7 (7.8)	
	1–2 weeks	148	43 (29.1)	
	3–5 days	45	25 (55.6)	
	Immediately before exam	20	18 (90.0)	
Usual sleep time	Before 24:00	155	23 (14.8)	<0.001
	24:00–01:00	111	34 (30.6)	
	01:00–02:00	45	30 (66.7)	
	After 02:00	11	10 (90.9)	
Breakfast frequency	Every day	42	1 (2.4)	<0.001
	Often	96	9 (9.4)	
	Sometimes	123	41 (33.3)	
	Never	61	46 (75.4)	
Breakfast timing	Before 07:00	15	4 (26.7)	<0.001
	07:00–08:00	203	40 (19.7)	
	08:00–09:00	33	6 (18.2)	
	After 09:00 or no breakfast	71	47 (66.2)	
Mobile-phone use (h/day)	<6 h	95	10 (10.5)	<0.001
	6–8 h	126	26 (20.6)	
	>8 h	101	61 (60.4)	
Gaming (h/day)	<2 h	124	9 (7.3)	<0.001
	2–4 h	23	2 (8.7)	
	4–6 h	143	59 (41.3)	
	>6 h	32	27 (84.4)	

Values are presented as *n* (%) unless otherwise indicated. *P*-values were obtained using Pearson’s chi-square test or Fisher’s exact test with Monte Carlo simulation, as appropriate, and are reported once for each characteristic. The complete descriptive results are provided in Supplementary Table 1.

### 2.3 Outcome definitions

The primary outcome, termed academic underperformance throughout, was GPA < 2.00, the passing threshold used for the analysis. Ninety-seven of 322 students met this definition. A secondary data-derived indicator, bottom-quartile GPA status, was defined as GPA at or below the empirical 25th percentile (1.929; 81 students). This secondary indicator was used only to describe cluster outcomes and was not treated as equivalent to academic underperformance.

### 2.4 Data collection and quality control

The questionnaire was administered through an online survey platform and completed by students using mobile phones in a classroom under instructions from trained researchers; completion took approximately 20 min. Of the 378 eligible students invited to participate, 56 did not complete the questionnaire. The remaining 322 students completed the survey and had complete information for the variables used in the analyses. Before statistical modeling, the analytical dataset was checked for category coding, outcome direction, denominators, and consistency across variables.

### 2.5 Association analyses

Categorical characteristics were summarized as counts and percentages. Associations with academic underperformance were screened descriptively using Pearson chi-square tests or Fisher exact tests with Monte Carlo simulation when sparse cells made chi-square approximations unsuitable. These tests were descriptive; multivariable covariate inclusion was not determined solely by univariable *P*-values.

Firth penalized logistic regression was used because sparse outcome cells produced complete or quasi-complete separation in several categories (Firth, 1993). For each behavior, crude and sex- and academic-year-adjusted odds ratios (ORs) with profile-likelihood 95% confidence intervals (CIs) were estimated. Two primary multivariable models were specified to address the primary study questions. Model A included breakfast frequency, daily gaming duration, sex, and academic year. Model B replaced breakfast frequency with breakfast timing. Breakfast frequency and timing were not entered together because skipping breakfast structurally determines the timing category for some students, creating conceptual overlap. Reference categories were reported explicitly.

Conventional logistic regression was used only as a sensitivity and diagnostic framework. Generalized variance inflation factors assessed multicollinearity. Area under the receiver-operating-characteristic curve (AUC), Brier score, the Hosmer–Lemeshow test, and Cook’s distance summarized model behavior; these quantities were not interpreted as external predictive validation. Effect modification was tested by adding all exposure-by-sex or exposure-by-academic-year interaction terms to the corresponding Firth model and comparing nested models. Stratified estimates were retained as exploratory supplementary analyses regardless of interaction significance. The inferential models did not explicitly account for potential within-class correlation arising from the class-based cluster sampling design.

### 2.6 Mixed-data cluster analysis

Eight routine variables were included in clustering: library use, online study, laboratory entry, review initiation, sleep time, breakfast frequency, mobile-phone use, and gaming duration. GPA, academic-underperformance indicators, sex, academic year, and breakfast timing were excluded. Excluding outcomes prevented circular profile construction; excluding breakfast timing avoided double-counting information already represented by breakfast frequency.

Gower dissimilarity was calculated for the mixed ordinal and categorical variables, followed by PAM. Candidate solutions from *k* = 2 to *k* = 6 were evaluated using average silhouette width. Stability was assessed with 200 repeated 80% subsamples; each subsample solution was mapped to the full-sample solution, and agreement was summarized using the adjusted Rand index (ARI). The selected solution maximized silhouette width while retaining high resampling stability. Profiles were named descriptively after inspecting their behavior distributions. GPA and the two outcome indicators were compared between profiles only after cluster formation using analysis of variance, the Kruskal–Wallis test, and chi-square tests, as appropriate.

All tests were two-sided with *P* < 0.05. Given the exploratory study and multiple comparisons, *P*-values were interpreted together with effect sizes, CIs, sparsity, and consistency across analyses. Analyses were performed in R version 4.5.1; the full analysis used a fixed random seed for resampling.

## 3 Results

### 3.1 Participant characteristics and outcome prevalence

Among 322 students, 97 (30.12%) had GPA < 2.00. Underperformance was observed in 35/168 female students (20.83%) and 62/154 male students (40.26%). Prevalence was 33.68% among freshmen, 38.18% among sophomores, and 19.66% among juniors. Descriptive gradients were also present across study time, laboratory entry, review timing, sleep, breakfast, mobile-phone use, and gaming categories (Table 1). These unadjusted comparisons describe the sample and do not establish independent effects.

### 3.2 Exposure-specific Firth associations

After adjustment for sex and academic year, several routine categories were associated with higher odds of academic underperformance (Table 2). Compared with eating breakfast every day, eating breakfast sometimes was associated with an aOR of 14.55 (95% CI: 3.50–134.90), and never eating breakfast with an aOR of 97.53 (95% CI: 21.18–952.97) in the exposure-specific model. Later sleep, shorter study time, no laboratory entry, late review initiation, longer mobile-phone use, and prolonged gaming also showed graded associations. Very wide CIs in the sparsest categories indicate substantial imprecision despite finite Firth estimates.

**TABLE 2:** Exposure-specific Firth logistic associations with academic underperformance.

Exposure	Category	Adjusted OR	95% CI	*P*
Library use (h/week)	>15 h (reference)	1.00	–	–
	7–15 h	2.82	0.60–27.31	0.210
	<7 h	14.62	3.68–132.95	<0.001
Online study (h/week)	>3 h (reference)	1.00	–	–
	1–3 h	8.31	2.64–41.68	<0.001
	<1 h	17.20	5.38–86.89	<0.001
Timing of first laboratory entry	Freshman (reference)	1.00	–	–
	Sophomore	0.90	0.27–2.79	0.850
	Junior	4.68	0.74–22.90	0.094
	Not yet entered	8.21	3.99–18.60	<0.001
Review initiation	Continuous (reference)	1.00	–	–
	1 month	0.39	0.10–1.60	0.182
	1–2 weeks	1.70	0.57–6.02	0.354
	3–5 days	4.99	1.51–19.34	0.008
	Immediately before exam	33.46	6.51–242.56	<0.001
Usual sleep time	Before 24:00 (reference)	1.00	–	–
	24:00–01:00	2.49	1.34–4.68	0.004
	01:00–02:00	9.29	4.24–21.20	<0.001
	After 02:00	36.96	7.49–368.90	<0.001
Breakfast frequency	Every day (reference)	1.00	–	–
	Often	3.02	0.65–28.99	0.173
	Sometimes	14.55	3.50–134.90	<0.001
	Never	97.53	21.17–952.97	<0.001
Breakfast timing	Before 07:00 (reference)	1.00	–	–
	07:00–08:00	0.61	0.19–2.20	0.422
	08:00–09:00	0.55	0.13–2.46	0.425
	After 09:00 or no breakfast	4.82	1.44–18.65	0.010
Mobile-phone use (h/day)	<6 h (reference)	1.00	–	–
	6–8 h	2.27	1.06–5.18	0.036
	>8 h	12.32	5.70–28.86	<0.001
Gaming (h/day)	<2 h (reference)	1.00	–	–
	2–4 h	1.64	0.29–6.58	0.534
	4–6 h	11.08	5.03–26.76	<0.001
	>6 h	84.02	25.26–326.33	<0.001

Each exposure was modeled separately using Firth penalized logistic regression with adjustment for sex and academic year. Reference categories are shown explicitly in the table. Adjusted ORs and profile-likelihood 95% CIs are reported. Full crude and adjusted results are provided in Supplementary Table 2. OR, odds ratio; CI, confidence interval.

### 3.3 Primary multivariable models and robustness checks

**FIGURE 1:**
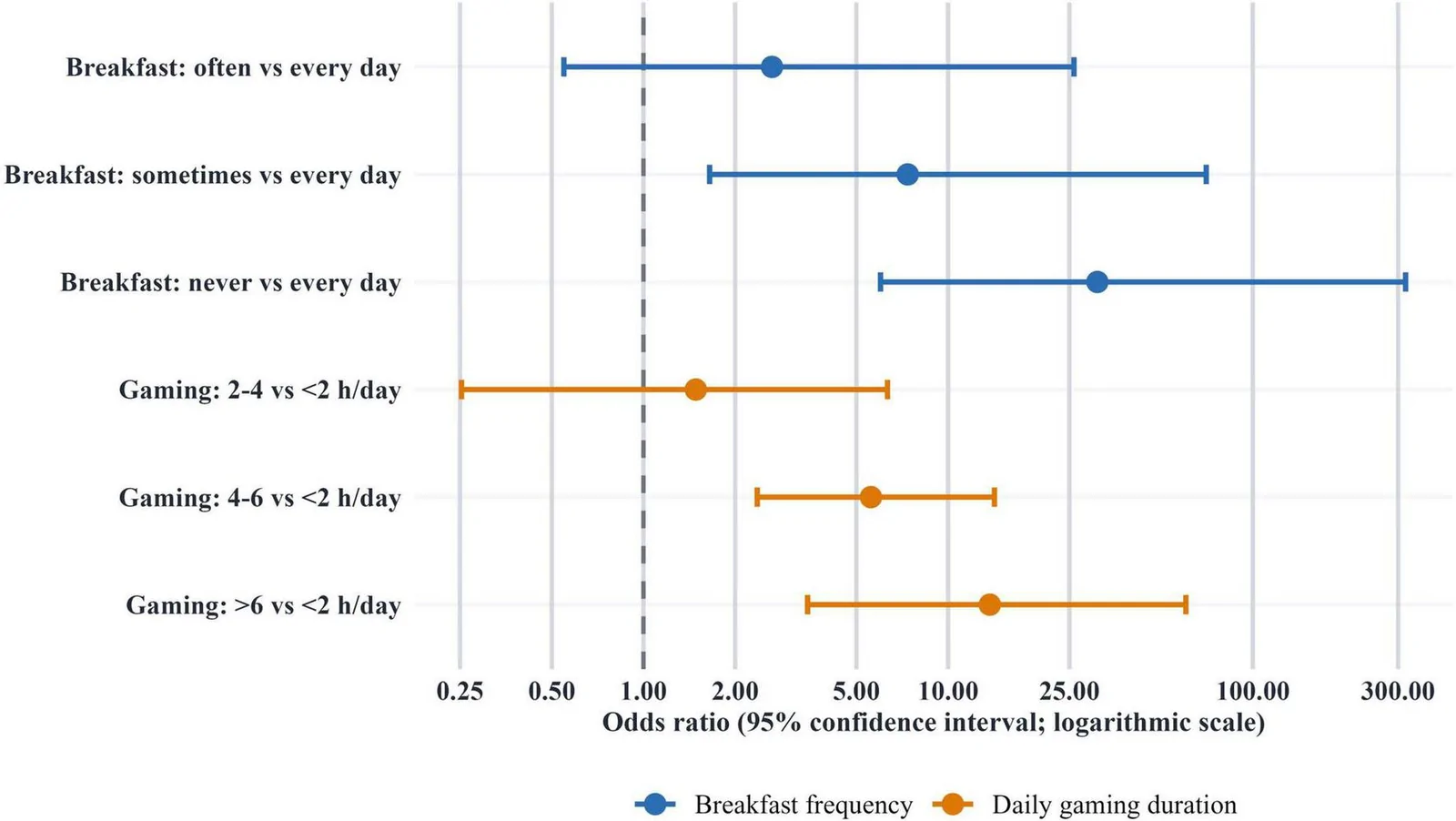
Adjusted associations of breakfast frequency and daily gaming duration with academic underperformance in primary Model A. Points represent adjusted odds ratios and horizontal lines represent 95% confidence intervals from Firth penalized logistic regression adjusted for sex and academic year. Blue indicates breakfast-frequency categories and orange indicates daily gaming-duration categories. The vertical dashed line denotes an odds ratio of 1. Reference categories were eating breakfast every day and gaming for <2 h/day. The horizontal axis is shown on a logarithmic scale.

In Model A, which included breakfast frequency and gaming duration, eating breakfast sometimes (aOR = 7.37, 95% CI: 1.65–70.32; *P* = 0.006) and never eating breakfast (aOR = 30.89, 95% CI: 6.00–317.35; *P* < 0.001) were associated with underperformance compared with eating breakfast every day. Gaming for 4–6 h/day (aOR = 5.58, 95% CI: 2.36–14.19; *P* < 0.001) and >6 h/day (aOR = 13.70, 95% CI: 3.46–60.29; *P* < 0.001) were associated with underperformance compared with <2 h/day (Figure 1 and Table 3). Sex was not independently associated after these adjustments, and the estimates for academic year were imprecise.

**TABLE 3:** Primary multivariable Firth logistic models for academic underperformance.

Model	Covariate/category	Adjusted OR	95% CI	*P*
Model A: breakfast frequency	Breakfast frequency: often	2.64	0.55–25.88	0.248
	Breakfast frequency: sometimes	7.37	1.65–70.32	0.006
	Breakfast frequency: never	30.89	6.00–317.35	<0.001
	Gaming: 2–4 h	1.49	0.25–6.32	0.627
	Gaming: 4–6 h	5.58	2.36–14.19	<0.001
	Gaming: >6 h	13.70	3.46–60.29	<0.001
	Sex: male	0.61	0.30–1.21	0.158
	Academic year: sophomore	1.92	0.92–4.06	0.081
	Academic year: junior	0.48	0.22–1.02	0.056
Model B: breakfast timing	Breakfast timing: 07:00–08:00	0.63	0.18–2.53	0.498
	Breakfast timing: 08:00–09:00	0.55	0.11–2.75	0.461
	Breakfast timing: after 09:00 or no breakfast	2.24	0.56–9.91	0.255
	Gaming: 2–4 h	1.68	0.30–6.87	0.515
	Gaming: 4–6 h	9.00	4.04–21.89	<0.001
	Gaming: >6 h	31.12	8.27–132.75	<0.001
	Sex: male	0.73	0.37–1.42	0.358
	Academic year: sophomore	1.78	0.87–3.72	0.116
	Academic year: junior	0.47	0.22–0.98	0.044

Model A included breakfast frequency, daily gaming duration, sex, and academic year. Model B included breakfast timing, daily gaming duration, sex, and academic year. Reference categories were eating breakfast every day for breakfast frequency, before 07:00 for breakfast timing, (2 h/day for gaming duration, female for sex, and freshman for academic year. Adjusted ORs and profile-likelihood 95% CIs were estimated using Firth penalized logistic regression. OR, odds ratio; CI, confidence interval.

In Model B, breakfast-timing categories were not independently associated with underperformance after adjustment for gaming, sex, and academic year. Gaming for 4–6 h/day (aOR = 9.00, 95% CI: 4.04–21.89) and >6 h/day (aOR = 31.12, 95% CI: 8.27–132.75) remained associated. The difference between the breakfast-frequency and breakfast-timing models supports treating the two constructs separately. Because the latest breakfast-timing category also included students who skipped breakfast to avoid sparse cells, this estimate should be interpreted cautiously.

The conventional-logistic sensitivity models had adjusted generalized variance-inflation factors of 1.03–1.16. Full model-diagnostic results are provided in Supplementary Table 3. Model A had AUC 0.877, Brier score 0.128, and Hosmer–Lemeshow *P* = 0.766; Model B had AUC 0.853, Brier score 0.138, and Hosmer–Lemeshow *P* = 0.748. Maximum Cook’s distances were 0.110 and 0.081, respectively. These diagnostics did not identify a single dominant multicollinearity or calibration concern, but they do not constitute out-of-sample validation. Global interaction tests were not significant for breakfast frequency, breakfast timing, or gaming by sex (*P* = 0.326, 0.893, and 0.377) or by academic year (*P* = 0.551, 0.732, and 0.701) (Supplementary Table 4A). Accordingly, sex-specific vulnerability and a distinct sophomore effect were not inferred; sparse stratified estimates are reported in Supplementary Table 4B.

### 3.4 Mixed-data behavioral profiles

**FIGURE 2:**
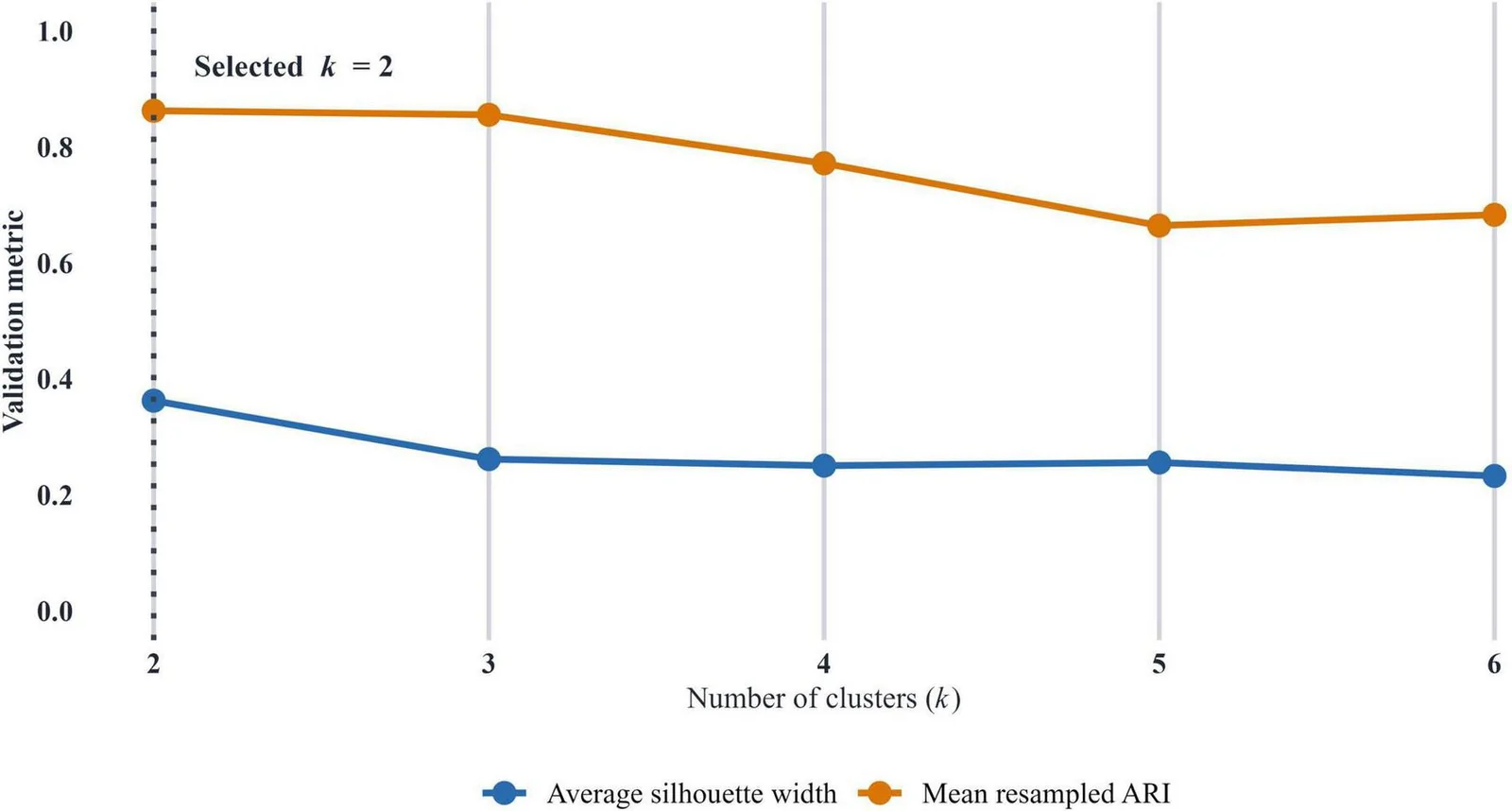
Validation of candidate PAM clustering solutions. Candidate solutions from *k* = 2 to *k* = 6 were evaluated using average silhouette width and resampling stability. Blue represents average silhouette width, and orange represents the mean adjusted Rand index (ARI) obtained from 200 repeated 80% subsamples. The vertical dotted line indicates the selected two-cluster solution. PAM, partitioning around medoids; ARI, adjusted Rand index.

Among *k* = 2–6 solutions, *k* = 2 had the largest average silhouette width (0.363) and high resampling stability (mean ARI 0.863; median 0.835; 10th–90th percentile 0.766–1.000) (Figure 2 and Supplementary Table 5). The two profiles were therefore retained. Cluster 1 (*n* = 182) was named the Dysregulated Routine profile and Cluster 2 (*n* = 140) the Structured Routine profile. These labels are descriptive rather than diagnostic.

**FIGURE 3:**
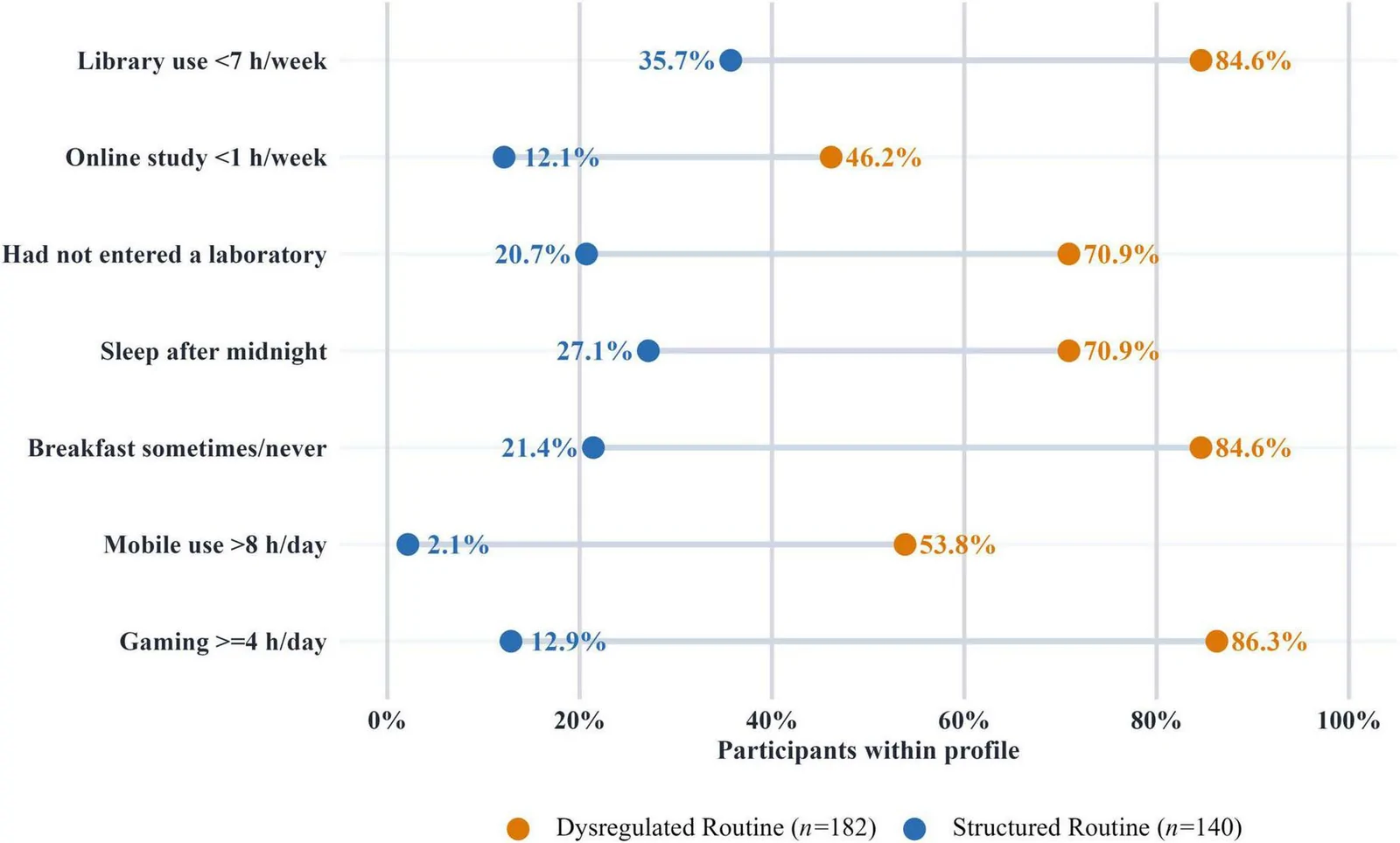
Selected behavioral characteristics of the Dysregulated Routine and Structured Routine profiles. Values represent the percentage of participants within each behavioral profile who met the corresponding behavioral criterion. Orange represents the Dysregulated Routine profile (*n* = 182), and blue represents the Structured Routine profile (*n* = 140). Behavioral characteristics were used to describe the clusters after partitioning around medoids (PAM) clustering and were not defined using grade point average (GPA) or academic-outcome information.

The Dysregulated Routine profile was characterized by library use <7 h/week (84.6%), online study <1 h/week (46.2%), no laboratory entry (70.9%), sleep after midnight (70.9%), breakfast sometimes or never (84.6%), mobile-phone use >8 h/day (53.8%), and gaming ≥4 h/day (86.3%). Corresponding percentages in the Structured Routine profile were 35.7%, 12.1%, 20.7%, 27.1%, 21.4%, 2.1%, and 12.9%, respectively (Figure 3).

**FIGURE 4:**
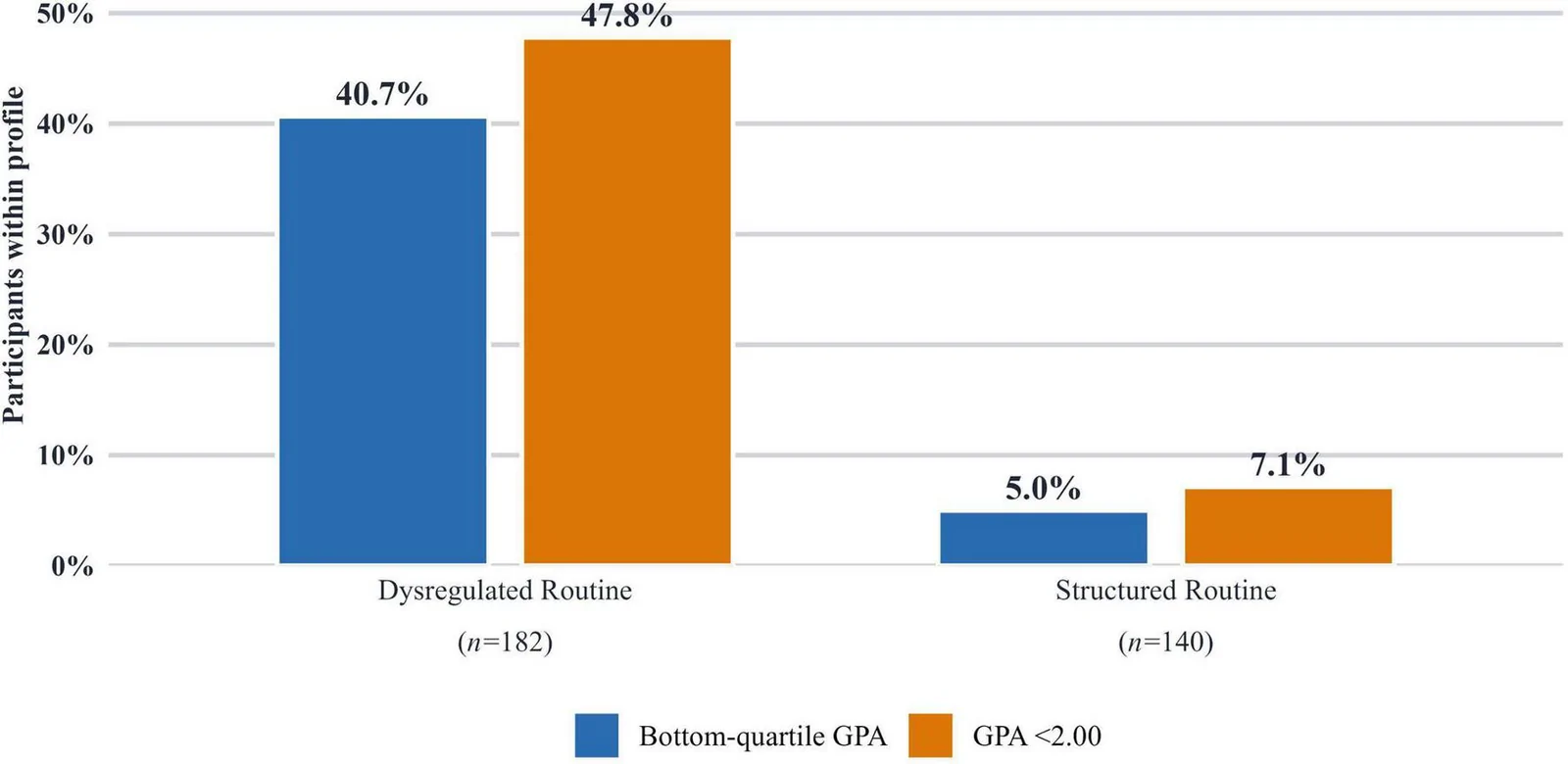
Academic outcomes across the Dysregulated Routine and Structured Routine profiles. Bars show the prevalence of academic underperformance, defined as grade point average (GPA) < 2.00, and bottom-quartile GPA status within each behavioral profile. Blue represents bottom-quartile GPA status and orange represents academic underperformance. GPA and both academic-outcome indicators were excluded from cluster formation and were compared between profiles only after the clusters had been derived.

Grade point average and outcome indicators were not used to form the clusters. Nevertheless, the Dysregulated Routine profile had a lower mean GPA (2.13, SD 0.63) than the Structured Routine profile (2.93, SD 0.58; analysis-of-variance *P* < 0.001; Kruskal–Wallis *P* < 0.001). Academic underperformance occurred in 87/182 students (47.8%) and 10/140 students (7.1%), respectively (chi-square *P* < 0.001). Bottom-quartile GPA status occurred in 74/182 (40.7%) and 7/140 (5.0%), respectively (chi-square *P* < 0.001) (Figure 4 and Table 4). These post-clustering comparisons describe separation of academic outcomes across profiles but do not validate individual prediction.

**TABLE 4:** Behavioral-profile characteristics and academic outcomes.

Characteristic/outcome	Dysregulated Routine, *n* (%)	Structured Routine, *n* (%)	*P*
Sample size	182	140	–
Library use < 7 h/week, *n* (%)	154 (84.6)	50 (35.7)	–
Online study < 1 h/week, *n* (%)	84 (46.2)	17 (12.1)	–
No laboratory entry, *n* (%)	129 (70.9)	29 (20.7)	–
Sleep after midnight, *n* (%)	129 (70.9)	38 (27.1)	–
Breakfast sometimes/never, *n* (%)	154 (84.6)	30 (21.4)	–
Mobile-phone use > 8 h/day, *n* (%)	98 (53.8)	3 (2.1)	–
Gaming ≥ 4 h/day, *n* (%)	157 (86.3)	18 (12.9)	–
GPA, mean (SD)	2.13 (0.63)	2.93 (0.58)	<0.001
GPA, median (IQR)	2.02 (0.86)	2.96 (0.68)	<0.001
GPA < 2.00, *n* (%)	87 (47.8)	10 (7.1)	<0.001
Bottom-quartile GPA, *n* (%)	74 (40.7)	7 (5.0)	<0.001

Values are presented as *n* (%) unless otherwise indicated. *P*-values were obtained using analysis of variance for mean GPA, the Kruskal–Wallis test for GPA distributions, and Pearson’s chi-square test for binary academic outcomes. Behavioral characteristics were used to describe the derived profiles, whereas GPA, academic underperformance, and bottom-quartile GPA status were assessed only after cluster formation and were not used to derive the clusters. SD, standard deviation; IQR, interquartile range; GPA, grade point average.

## 4 Discussion

In this cross-sectional sample of 322 life-sciences undergraduates, academic underperformance, defined as GPA < 2.00, was observed in 30.12% of students. In the primary multivariable Firth models, lower breakfast frequency and prolonged daily gaming remained associated with academic underperformance after adjustment for sex, academic year, and the co-modeled behavioral factor. Formal interaction tests provided no evidence that these associations differed by sex or academic year. Mixed-data clustering, performed without GPA or academic-outcome indicators, identified two routine profiles with markedly different academic outcomes.

Breakfast consumption has been linked to attention, memory, and academic outcomes in student populations (Cheng and Rebecca Yew, 2025; Gao et al., 2021; López-Gil et al., 2022; Regan et al., 2023). In our primary frequency model, the odds of underperformance rose across the sometimes and never categories, but the CIs were wide, particularly for students who never ate breakfast. This pattern is consistent with an association between breakfast regularity and academic status, but the data cannot establish that breakfast changes GPA. Breakfast timing was not independently associated in the separate timing model, underscoring the importance of distinguishing meal frequency from clock time and avoiding a combined “late or skipped” exposure.

Longer gaming duration also remained associated with underperformance in both primary models. This finding is compatible with research linking problematic digital-media use, sleep disruption, and academic achievement (Ou-Yang et al., 2023; Paterna et al., 2024; Pyhältö et al., 2025; Rathakrishnan et al., 2021; Zhou et al., 2022), yet daily gaming may also mark unmeasured factors such as stress, disengagement, mental health, or time-allocation constraints. Sex was not independently associated in the primary models, and interaction tests did not indicate that the gaming association differed by sex or academic year. The subgroup estimates therefore should not be interpreted as evidence of a male vulnerability or a sophomore slump.

The two-profile solution summarized co-occurring routines using an approach suited to mixed ordinal and categorical variables. Gower dissimilarity accommodates mixed data types, while PAM represents clusters using observed medoids. The Dysregulated Routine profile combined shorter study time, delayed sleep, irregular breakfast, and greater mobile-phone and gaming time. Its lower GPA and higher underperformance prevalence provide convergent descriptive evidence, but the moderate silhouette width, single sample, and post-clustering outcome comparisons mean that the profiles are exploratory. They should not be used as diagnostic labels or automated risk scores without prospective external validation.

For student-support practice, the results suggest several testable targets for low-burden assessment, including meal regularity, gaming duration, sleep timing, and study routines, rather than a ready-to-deploy surveillance system. Institutions could evaluate voluntary, non-stigmatizing support packages that combine academic coaching, time-management resources, sleep and nutrition education, and referral pathways (Muriago et al., 2026). Such approaches are consistent with emerging evidence from university-based health-behavior and sleep interventions, although the effectiveness and implementation of multicomponent programs remain heterogeneous (Chandler et al., 2022; Streram et al., 2025). Any future screening tool should be prospectively validated, assessed for calibration and fairness, and implemented with informed governance rather than inferred directly from the present ORs or clusters.

Several limitations warrant caution. The cross-sectional design does not establish temporal ordering or causality. The single-institution sampling frame limits generalizability, and non-response may have introduced selection bias. Although classes were randomly selected, the study was conducted within a single school, and students who did not complete the questionnaire may have differed systematically from those included in the final analytical sample. In addition, because classes served as the sampling units, observations within the same class may have been correlated. As the inferential models did not explicitly account for this clustering, standard errors may have been underestimated and confidence intervals may have been too narrow. Behaviors were self-reported and may also be affected by recall or social-desirability bias, while residual confounding by mental health, course load, socioeconomic context, and other determinants remains possible. Some categories contained few outcome events, producing wide CIs even with Firth estimation. Multiple exposure comparisons were exploratory. Conventional-model diagnostics were in-sample, not external validation. Finally, the cluster labels and the empirical bottom-quartile cutoff were data-derived and require replication in independent cohorts.

## 5 Conclusion

Lower breakfast frequency and prolonged daily gaming were associated with academic underperformance among life-sciences undergraduates, and two routine profiles showed markedly different academic outcomes. These findings support the value of examining co-occurring daily behaviors, but they should be interpreted as exploratory and non-causal. Prospective studies in independent populations are needed to determine whether these behavioral patterns can meaningfully inform student-support strategies.

## Data Availability

The datasets presented in this article are not readily available because they contain sensitive student-level information and are subject to privacy and ethical restrictions. De-identified data may be made available upon reasonable request to the corresponding author, subject to applicable ethical, institutional, and privacy requirements. Requests to access the datasets should be directed to Ping Zeng (zpstat@xzhmu.edu.cn) or Chu Zheng (zhengchufory@xzhmu.edu.cn).
