# Supplementary material for: Daily behavioral profiles and academic underperformance among university students: a cross-sectional study
Source: Front Psychol. 2026 Sep 11;17:1856492. doi: 10.3389/fpsyg.2026.1856492 (PMC13612416; doi:10.3389/fpsyg.2026.1856492)
Supplement: Supplementary file 1 [file Supplementary_file_1.docx]

Supplementary File

Table S1. Complete descriptive characteristics by academic-underperformance status (GPA <2.00).

| Characteristic | Category | Total *n* | GPA <2.00, *n* (%) | *P* |
| --- | --- | --- | --- | --- |
| Sex | Female | 168 | 35 (20.8) | <0.001 |
|  | Male | 154 | 62 (40.3) |  |
| Academic year | Freshman | 95 | 32 (33.7) | 0.007 |
|  | Sophomore | 110 | 42 (38.2) |  |
|  | Junior | 117 | 23 (19.7) |  |
| Political status | Mass | 145 | 53 (36.6) | 0.025 |
|  | League member | 170 | 44 (25.9) |  |
|  | Probationary party member | 7 | 0 (0.0) |  |
| Family structure | Non-single-parent | 291 | 84 (28.9) | 0.132 |
|  | Single-parent | 31 | 13 (41.9) |  |
| Father's education | Junior high or below | 124 | 39 (31.5) | 0.448 |
|  | High school | 93 | 26 (28.0) |  |
|  | Junior college | 41 | 16 (39.0) |  |
|  | Bachelor or above | 64 | 16 (25.0) |  |
| Mother's education | Junior high or below | 153 | 50 (32.7) | 0.414 |
|  | High school | 72 | 21 (29.2) |  |
|  | Junior college | 52 | 17 (32.7) |  |
|  | Bachelor or above | 45 | 9 (20.0) |  |
| Perceived family economy | No financial pressure | 118 | 37 (31.4) | 0.623 |
|  | Slight pressure | 161 | 45 (28.0) |  |
|  | High pressure | 34 | 13 (38.2) |  |
|  | External support required | 9 | 2 (22.2) |  |
| Library use (h/week) | >15 h | 38 | 1 (2.6) | <0.001 |
|  | 7–15 h | 80 | 9 (11.2) |  |
|  | <7 h | 204 | 87 (42.6) |  |
| Online study (h/week) | >3 h | 62 | 2 (3.2) | <0.001 |
|  | 1–3 h | 159 | 45 (28.3) |  |
|  | <1 h | 101 | 50 (49.5) |  |
| Timing of first laboratory entry | Freshman | 90 | 9 (10.0) | <0.001 |
|  | Sophomore | 62 | 5 (8.1) |  |
|  | Junior | 12 | 2 (16.7) |  |
|  | Not yet entered | 158 | 81 (51.3) |  |
| Review initiation | Continuous | 19 | 4 (21.1) | <0.001 |
|  | One month | 90 | 7 (7.8) |  |
|  | 1–2 weeks | 148 | 43 (29.1) |  |
|  | 3–5 days | 45 | 25 (55.6) |  |
|  | Immediately before exam | 20 | 18 (90.0) |  |
| Usual sleep time | Before 24:00 | 155 | 23 (14.8) | <0.001 |
|  | 24:00–01:00 | 111 | 34 (30.6) |  |
|  | 01:00–02:00 | 45 | 30 (66.7) |  |
|  | After 02:00 | 11 | 10 (90.9) |  |
| Breakfast frequency | Every day | 42 | 1 (2.4) | <0.001 |
|  | Often | 96 | 9 (9.4) |  |
|  | Sometimes | 123 | 41 (33.3) |  |
|  | Never | 61 | 46 (75.4) |  |
| Breakfast timing | Before 07:00 | 15 | 4 (26.7) | <0.001 |
|  | 07:00–08:00 | 203 | 40 (19.7) |  |
|  | 08:00–09:00 | 33 | 6 (18.2) |  |
|  | After 09:00 or no breakfast | 71 | 47 (66.2) |  |
| Mobile-phone use (h/day) | <6 h | 95 | 10 (10.5) | <0.001 |
|  | 6–8 h | 126 | 26 (20.6) |  |
|  | >8 h | 101 | 61 (60.4) |  |
| Gaming (h/day) | <2 h | 124 | 9 (7.3) | <0.001 |
|  | 2–4 h | 23 | 2 (8.7) |  |
|  | 4–6 h | 143 | 59 (41.3) |  |
|  | >6 h | 32 | 27 (84.4) |  |
| Career plan clarity | Very clear | 20 | 2 (10.0) | <0.001 |
|  | Basically clear | 160 | 28 (17.5) |  |
|  | Rather confused | 138 | 65 (47.1) |  |
|  | Very confused | 4 | 2 (50.0) |  |
| Further-study intention | Clear plan | 117 | 22 (18.8) | 0.002 |
|  | Interested but undecided | 138 | 45 (32.6) |  |
|  | Not considering for now | 51 | 23 (45.1) |  |
|  | Not considering | 16 | 7 (43.8) |  |
| Attitude toward major | Like | 206 | 37 (18.0) | <0.001 |
|  | Neutral | 109 | 54 (49.5) |  |
|  | Dislike | 7 | 6 (85.7) |  |
| Professional identity | Agree | 208 | 38 (18.3) | <0.001 |
|  | Rather confused | 108 | 55 (50.9) |  |
|  | Pessimistic | 6 | 4 (66.7) |  |

***Note:*** *Values are presented as n (%) unless otherwise indicated. P values are descriptive and unadjusted and were obtained using Pearson's chi-square test or Fisher's exact test with Monte Carlo simulation, as appropriate.*

Table S2. Crude and adjusted exposure-specific Firth logistic regression results.

| Exposure | Category | Crude OR | 95% CI | *P* | Adjusted OR | 95% CI | *P* |
| --- | --- | --- | --- | --- | --- | --- | --- |
| Library use (h/week) | >15 h (reference) | 1.00 | — | — | 1.00 | — | — |
|  | 7–15 h | 3.32 | 0.72–31.80 | 0.133 | 2.82 | 0.60–27.31 | 0.210 |
|  | <7 h | 18.62 | 4.82–167.43 | <0.001 | 14.62 | 3.68–132.95 | <0.001 |
| Online study (h/week) | >3 h (reference) | 1.00 | — | — | 1.00 | — | — |
|  | 1–3 h | 9.62 | 3.11–47.80 | <0.001 | 8.31 | 2.64–41.68 | <0.001 |
|  | <1 h | 23.73 | 7.56–118.97 | <0.001 | 17.20 | 5.38–86.89 | <0.001 |
| Timing of first laboratory entry | Freshman (reference) | 1.00 | — | — | 1.00 | — | — |
|  | Sophomore | 0.82 | 0.25–2.41 | 0.724 | 0.90 | 0.27–2.79 | 0.850 |
|  | Junior | 2.04 | 0.35–8.59 | 0.385 | 4.68 | 0.74–22.90 | 0.094 |
|  | Not yet entered | 9.02 | 4.49–20.03 | <0.001 | 8.21 | 3.99–18.60 | <0.001 |
| Review initiation | Continuous (reference) | 1.00 | — | — | 1.00 | — | — |
|  | One month | 0.31 | 0.09–1.21 | 0.088 | 0.39 | 0.10–1.60 | 0.182 |
|  | 1–2 weeks | 1.42 | 0.50–4.84 | 0.525 | 1.70 | 0.57–6.02 | 0.354 |
|  | 3–5 days | 4.28 | 1.37–15.77 | 0.011 | 4.99 | 1.51–19.34 | 0.008 |
|  | Immediately before exam | 25.49 | 5.44–171.78 | <0.001 | 33.46 | 6.51–242.56 | <0.001 |
| Usual sleep time | Before 24:00 (reference) | 1.00 | — | — | 1.00 | — | — |
|  | 24:00–01:00 | 2.51 | 1.39–4.59 | 0.002 | 2.49 | 1.34–4.68 | 0.004 |
|  | 01:00–02:00 | 11.09 | 5.32–24.12 | <0.001 | 9.29 | 4.24–21.20 | <0.001 |
|  | After 02:00 | 39.47 | 8.65–378.15 | <0.001 | 36.96 | 7.49–368.90 | <0.001 |
| Breakfast frequency | Every day (reference) | 1.00 | — | — | 1.00 | — | — |
|  | Often | 3.00 | 0.66–28.66 | 0.171 | 3.02 | 0.65–28.99 | 0.173 |
|  | Sometimes | 13.92 | 3.52–126.29 | <0.001 | 14.55 | 3.50–134.90 | <0.001 |
|  | Never | 83.00 | 19.49–779.30 | <0.001 | 97.53 | 21.17–952.97 | <0.001 |
| Breakfast timing | Before 07:00 (reference) | 1.00 | — | — | 1.00 | — | — |
|  | 07:00–08:00 | 0.63 | 0.21–2.21 | 0.446 | 0.61 | 0.19–2.20 | 0.422 |
|  | 08:00–09:00 | 0.60 | 0.15–2.55 | 0.480 | 0.55 | 0.13–2.46 | 0.425 |
|  | After 09:00 or no breakfast | 4.95 | 1.58–18.09 | 0.005 | 4.82 | 1.44–18.65 | 0.010 |
| Mobile-phone use (h/day) | <6 h (reference) | 1.00 | — | — | 1.00 | — | — |
|  | 6–8 h | 2.15 | 1.02–4.83 | 0.044 | 2.27 | 1.06–5.18 | 0.036 |
|  | >8 h | 12.37 | 6.03–27.52 | <0.001 | 12.32 | 5.70–28.86 | <0.001 |
| Gaming (h/day) | <2 h (reference) | 1.00 | — | — | 1.00 | — | — |
|  | 2–4 h | 1.41 | 0.26–5.45 | 0.653 | 1.64 | 0.29–6.58 | 0.534 |
|  | 4–6 h | 8.56 | 4.26–18.99 | <0.001 | 11.08 | 5.03–26.76 | <0.001 |
|  | >6 h | 60.79 | 21.11–206.94 | <0.001 | 84.02 | 25.26–326.33 | <0.001 |
| Career plan clarity | Very clear (reference) | 1.00 | — | — | 1.00 | — | — |
|  | Basically clear | 1.59 | 0.47–8.28 | 0.489 | 1.97 | 0.56–10.48 | 0.314 |
|  | Rather confused | 6.59 | 1.98–33.89 | 0.001 | 7.13 | 2.08–37.31 | <0.001 |
|  | Very confused | 7.40 | 0.82–77.71 | 0.074 | 9.12 | 0.89–108.98 | 0.062 |
| Further-study intention | Clear plan (reference) | 1.00 | — | — | 1.00 | — | — |
|  | Interested but undecided | 2.07 | 1.17–3.74 | 0.013 | 1.76 | 0.96–3.26 | 0.066 |
|  | Not considering for now | 3.50 | 1.72–7.21 | <0.001 | 3.00 | 1.41–6.42 | 0.004 |
|  | Not considering | 3.35 | 1.13–9.72 | 0.030 | 1.65 | 0.52–5.11 | 0.389 |
| Attitude toward major | Like (reference) | 1.00 | — | — | 1.00 | — | — |
|  | Neutral | 4.44 | 2.67–7.48 | <0.001 | 4.67 | 2.71–8.20 | <0.001 |
|  | Dislike | 19.59 | 3.96–192.00 | <0.001 | 14.56 | 2.77–146.88 | 0.001 |
| Professional identity | Agree (reference) | 1.00 | — | — | 1.00 | — | — |
|  | Rather confused | 4.59 | 2.76–7.73 | <0.001 | 4.53 | 2.63–7.94 | <0.001 |
|  | Pessimistic | 7.97 | 1.70–47.11 | 0.009 | 9.40 | 1.82–60.37 | 0.008 |

***Note:*** *Crude models included each exposure separately. Adjusted models included the corresponding exposure together with sex and academic year. Odds ratios and profile-likelihood 95% confidence intervals were estimated using Firth penalized logistic regression. Reference categories are indicated in the table. OR, odds ratio; CI, confidence interval.*

Table S3. Multicollinearity and conventional-model diagnostics.

| Model | Term | GVIF | df | Adjusted GVIF |
| --- | --- | --- | --- | --- |
| Model A: breakfast frequency | Breakfast frequency | 1.575 | 3 | 1.079 |
| Model A: breakfast frequency | Gaming duration | 1.796 | 3 | 1.102 |
| Model A: breakfast frequency | Sex | 1.347 | 1 | 1.161 |
| Model A: breakfast frequency | Academic year | 1.122 | 2 | 1.029 |
| Model B: breakfast timing | Breakfast timing | 1.415 | 3 | 1.060 |
| Model B: breakfast timing | Gaming duration | 1.621 | 3 | 1.084 |
| Model B: breakfast timing | Sex | 1.337 | 1 | 1.156 |
| Model B: breakfast timing | Academic year | 1.158 | 2 | 1.037 |

| Model | AUC | Brier score | Hosmer-Lemeshow *P* | Maximum Cook's D | Cook's D >4/*n* |
| --- | --- | --- | --- | --- | --- |
| Model A: breakfast frequency | 0.877 | 0.128 | 0.766 | 0.110 | 28 |
| Model B: breakfast timing | 0.853 | 0.138 | 0.748 | 0.081 | 23 |

***Note:*** *Diagnostics were obtained from conventional logistic regression models fitted as sensitivity analyses and were not interpreted as measures of external predictive validity. Adjusted GVIF denotes GVIF^1/(2×df)^. Cook's D >4/n indicates the number of observations with Cook's distance greater than 4/n. GVIF, generalized variance inflation factor; df, degrees of freedom; AUC, area under the receiver operating characteristic curve.*

Table S4A. Global interaction tests for sex and academic year.

| Global interaction | Likelihood-ratio *P* |
| --- | --- |
| Breakfast frequency x sex | 0.326 |
| Breakfast timing x sex | 0.893 |
| Gaming duration x sex | 0.377 |
| Breakfast frequency x academic year | 0.551 |
| Breakfast timing x academic year | 0.732 |
| Gaming duration x academic year | 0.701 |

***Note:*** *Global likelihood-ratio tests were used to assess evidence of effect modification. The interaction tests, rather than differences in statistical significance between strata, were used for interpretation.*

Table S4B. Exploratory sex- and academic-year-stratified Firth logistic regression estimates.

| Stratifier | Level | Exposure | Category | *Stratum N*/Events | OR (95% CI) | *P* |
| --- | --- | --- | --- | --- | --- | --- |
| Sex | Female | Breakfast frequency | Every day (reference) | 168/35 | 1.00 | — |
|  |  |  | Often |  | 2.29 (0.45–22.59) | 0.341 |
|  |  |  | Sometimes |  | 14.28 (3.31–134.18) | <0.001 |
|  |  |  | Never |  | 28.29 (5.32–294.93) | <0.001 |
| Sex | Female | Breakfast timing | Before 07:00 (reference) | 168/35 | 1.00 | — |
|  |  |  | 07:00–08:00 |  | 0.47 (0.13–2.05) | 0.291 |
|  |  |  | 08:00–09:00 |  | 0.56 (0.11–3.00) | 0.485 |
|  |  |  | After 09:00 or no breakfast |  | 2.22 (0.52–11.07) | 0.288 |
| Sex | Female | Gaming (h/day) | <2 h (reference) | 168/35 | 1.00 | — |
|  |  |  | 2–4 h |  | 2.65 (0.46–11.29) | 0.248 |
|  |  |  | 4–6 h |  | 7.94 (3.43–19.66) | <0.001 |
|  |  |  | >6 h |  | 16.75 (2.02–197.84) | 0.011 |
| Sex | Male | Breakfast frequency | Every day (reference) | 154/62 | 1.00 | — |
|  |  |  | Often |  | 2.43 (0.20–339.44) | 0.534 |
|  |  |  | Sometimes |  | 7.29 (0.84–956.92) | 0.077 |
|  |  |  | Never |  | 75.00 (8.03–10056.20) | <0.001 |
| Sex | Male | Breakfast timing | Before 07:00 (reference) | 154/62 | 1.00 | — |
|  |  |  | 07:00–08:00 |  | 0.77 (0.12–8.21) | 0.795 |
|  |  |  | 08:00–09:00 |  | 0.69 (0.06–9.54) | 0.758 |
|  |  |  | After 09:00 or no breakfast |  | 6.48 (0.96–71.47) | 0.055 |
| Sex | Male | Gaming (h/day) | <2 h (reference) | 154/62 | 1.00 | — |
|  |  |  | 2–4 h |  | 1.64 (0.01–312.20) | 0.809 |
|  |  |  | 4–6 h |  | 27.21 (3.55–3498.74) | <0.001 |
|  |  |  | >6 h |  | 232.33 (24.37–31589.16) | <0.001 |
| Academic year | Freshman | Breakfast frequency | Every day (reference) | 95/32 | 1.00 | — |
|  |  |  | Often |  | 5.37 (0.52–730.24) | 0.182 |
|  |  |  | Sometimes |  | 8.47 (0.94–1122.96) | 0.058 |
|  |  |  | Never |  | 77.00 (7.47–10643.06) | <0.001 |
| Academic year | Freshman | Breakfast timing | Before 07:00 (reference) | 95/32 | 1.00 | — |
|  |  |  | 07:00–08:00 |  | 0.61 (0.16–2.81) | 0.504 |
|  |  |  | 08:00–09:00 |  | 1.35 (0.17–10.10) | 0.768 |
|  |  |  | After 09:00 or no breakfast |  | 6.16 (1.41–32.48) | 0.015 |
| Academic year | Freshman | Gaming (h/day) | <2 h (reference) | 95/32 | 1.00 | — |
|  |  |  | 2–4 h |  | 5.91 (0.45–60.71) | 0.156 |
|  |  |  | 4–6 h |  | 6.88 (2.17–28.26) | <0.001 |
|  |  |  | >6 h |  | 69.00 (11.45–805.72) | <0.001 |
| Academic year | Sophomore | Breakfast frequency | Every day (reference) | 110/42 | 1.00 | — |
|  |  |  | Often |  | 1.11 (0.17–12.20) | 0.916 |
|  |  |  | Sometimes |  | 11.39 (2.45–110.49) | <0.001 |
|  |  |  | Never |  | 72.33 (11.05–934.59) | <0.001 |
| Academic year | Sophomore | Breakfast timing | Before 07:00 (reference) | 110/42 | 1.00 | — |
|  |  |  | 07:00–08:00 |  | 2.03 (0.16–284.72) | 0.629 |
|  |  |  | 08:00–09:00 |  | 1.67 (0.11–246.96) | 0.746 |
|  |  |  | After 09:00 or no breakfast |  | 26.43 (1.69–3979.01) | 0.018 |
| Academic year | Sophomore | Gaming (h/day) | <2 h (reference) | 110/42 | 1.00 | — |
|  |  |  | 2–4 h |  | 1.59 (0.15–10.18) | 0.658 |
|  |  |  | 4–6 h |  | 13.38 (4.64–47.04) | <0.001 |
|  |  |  | >6 h |  | 37.80 (8.02–254.76) | <0.001 |
| Academic year | Junior | Breakfast frequency | Every day (reference) | 117/23 | 1.00 | — |
|  |  |  | Often |  | 1.43 (0.07–214.04) | 0.823 |
|  |  |  | Sometimes |  | 8.83 (1.01–1163.66) | 0.049 |
|  |  |  | Never |  | 46.89 (5.16–6254.13) | <0.001 |
| Academic year | Junior | Breakfast timing | Before 07:00 (reference) | 117/23 | 1.00 | — |
|  |  |  | 07:00–08:00 |  | 0.14 (0.01–1.84) | 0.121 |
|  |  |  | 08:00–09:00 |  | 0.05 (0.00–1.30) | 0.071 |
|  |  |  | After 09:00 or no breakfast |  | 0.87 (0.06–11.70) | 0.909 |
| Academic year | Junior | Gaming (h/day) | <2 h (reference) | 117/23 | 1.00 | — |
|  |  |  | 2–4 h |  | 0.72 (0.01–9.73) | 0.832 |
|  |  |  | 4–6 h |  | 5.94 (1.68–31.55) | 0.004 |
|  |  |  | >6 h |  | 49.80 (8.59–435.47) | <0.001 |

***Note:*** *Stratified estimates were obtained using Firth penalized logistic regression and are presented as exploratory analyses. Several strata contained few outcome events, resulting in wide profile-likelihood confidence intervals. Evidence of effect modification was assessed using the global interaction tests in Supplementary Table S4A rather than by comparing statistical significance across strata. OR, odds ratio; CI, confidence interval.*

Table S5. PAM cluster validation for *k*=2–6.

| *k* | Average silhouette width | Mean ARI | Median ARI | ARI 10th percentile | ARI 90th percentile |
| --- | --- | --- | --- | --- | --- |
| 2 | 0.363 | 0.863 | 0.835 | 0.766 | 1.000 |
| 3 | 0.262 | 0.856 | 0.883 | 0.704 | 1.000 |
| 4 | 0.251 | 0.772 | 0.824 | 0.492 | 1.000 |
| 5 | 0.256 | 0.665 | 0.618 | 0.522 | 0.886 |
| 6 | 0.233 | 0.683 | 0.684 | 0.496 | 0.869 |

***Note:*** *Candidate solutions from k=2 to k=6 were evaluated using average silhouette width and resampling stability. Stability was assessed using 200 repeated 80% subsamples and summarized using the adjusted Rand index (ARI). The k=2 solution was retained because it had the largest average silhouette width while maintaining high resampling stability. GPA and both academic-outcome indicators were excluded from cluster formation. ARI, adjusted Rand index.*

Table S6. Mapping of original questionnaire responses to analytic categories.

| Variable | Original questionnaire categories | Analytic categories |
| --- | --- | --- |
| Library use (h/week) | <7 h; 7–15 h; 15–30 h; >30 h | <7 h; 7–15 h; >15 h (15–30 h and >30 h combined) |
| Online study (h/week) | <1 h; 1–3 h; 3–6 h; >6 h | <1 h; 1–3 h; >3 h (3–6 h and >6 h combined) |
| Timing of first laboratory entry | First or second semester of freshman, sophomore, or junior year; during senior-year internship; not yet entered | Freshman; Sophomore; Junior; not yet entered. Semester-specific categories were combined within academic year. The senior-year internship category had no observations and was not represented in the analytic dataset. |
| Usual sleep time | Before 23:00; 23:00–24:00; 24:00–01:00; 01:00–02:00; after 02:00 | Before 24:00 (before 23:00 and 23:00–24:00 combined); 24:00–01:00; 01:00–02:00; after 02:00 |
| Breakfast timing | Before 07:00; 07:00–08:00; 08:00–09:00; after 09:00; no breakfast | Before 07:00; 07:00–08:00; 08:00–09:00; after 09:00 or no breakfast (last two categories combined) |
| Mobile-phone use (h/day) | <4 h; 4–6 h; 6–8 h; >8 h | <6 h (<4 h and 4–6 h combined); 6–8 h; >8 h |

***Note:*** *Adjacent response categories were combined before analysis to reduce sparse cells and improve interpretability. Categories not listed in this table were retained as collected, apart from English-language relabeling for reporting. h, hours.*
